# Evaluating organochlorine pesticide residues in the aquatic environment of the Lake Naivasha River basin using passive sampling techniques

**DOI:** 10.1007/s10661-018-6713-4

**Published:** 2018-05-18

**Authors:** Yasser Abbasi, Chris M. Mannaerts

**Affiliations:** Department of Water Resources (WRS), Faculty of Geo-information Sciences and Earth Observation (ITC), University of Twente (UT), P.O. Box 217, 7500AE, Enschede, The Netherlands

**Keywords:** Pesticide residues, Passive sampling, Silicone rubber sheet, Speedisk, Lake Naivasha

## Abstract

Passive sampling techniques can improve the discovery of low concentrations by continuous collecting the contaminants, which usually go undetected with classic and once-off time-point grab sampling. The aim of this study was to evaluate organochlorine pesticide (OCP) residues in the aquatic environment of the Lake Naivasha river basin (Kenya) using passive sampling techniques. Silicone rubber sheet and Speedisk samplers were used to detect residues of α-HCH, β-HCH, γ-HCH, δ-HCH, heptachlor, aldrin, heptachlor epoxide, pp-DDE, endrin, dieldrin, α-endosulfan, β-endosulfan, pp-DDD, endrin aldehyde, pp-DDT, endosulfan sulfate, and methoxychlor in the Malewa River and Lake Naivasha. After solvent extraction from the sampling media, the residues were analyzed using gas chromatography electron capture detection (GC-ECD) for the OCPs and gas chromatography-mass spectrometry (GC-MS) for the PCB reference compounds. Measuring the OCP residues using the silicone rubber samplers revealed the highest concentration of residues (∑OCPs of 81 (± 18.9 SD) μg/L) to be at the Lake site, being the ultimate accumulation environment for surficial hydrological, chemical, and sediment transport through the river basin. The total OCP residue sums changed to 71.5 (± 11.3 SD) μg/L for the Middle Malewa and 59 (± 12.5 SD) μg/L for the Upper Malewa River sampling sites. The concentration sums of OCPs detected using the Speedisk samplers at the Upper Malewa, Middle Malewa, and the Lake Naivasha sites were 28.2 (± 4.2 SD), 31.3 (± 1.8 SD), and 34.2 (± 6.4 SD) μg/L, respectively. An evaluation of the different pesticide compound variations identified at the three sites revealed that endosulfan sulfate, α-HCH, methoxychlor, and endrin aldehyde residues were still found at all sampling sites. However, the statistical analysis of one-way ANOVA for testing the differences of ∑OCPs between the sampling sites for both the silicone rubber sheet and Speedisk samplers showed that there was no significant difference from the Upper Malewa to the Lake site (*P* < 0.05). Finally, the finding of this study indicated that continued monitoring of pesticides residues in the catchment remains highly recommended.

## Introduction

The first application of organochlorine pesticides such as DDT and dieldrin dates back to 1956 and 1961, respectively, but due to the long half-life and their bioaccumulation in animal body, they were banned globally in 1976 (Keating 1983), except for regulated use of DDT for the control of malaria. After application of the chemicals, their residues can reach non-targets such as plants, soil, water, and sediment, by which these environmental compartments could be contaminated. Kaoga et al. (2013) explained that over 95% of applied insecticides and herbicides end up in non-target areas. This could potentially endanger the environment and also contribute to public health problems (Mutuku et al. 2014). Although most of the pesticides have a short half-life and are easily degradable, there are still persistent pesticides such as first-generation organochlorine pesticides (OCPs), which have long time half-life and are persistent in environment. Consequently, they can be washed off to water bodies and cause considerable environmental risk (Gitahi et al. 2002). Lakes and reservoirs are typical accumulation sites for runoff, sediment, and chemicals in catchments, and therefore, these aquatic environments are at risk of being contaminated. Bearing in mind that due to the threat of pesticides, residues pose to aquatic life and ecosystems, careful evaluation is needed.

Aquatic monitoring programs are usually based on grab samples collected within a short time span. Grab sampling can only provide a snapshot of pollution levels (Hernando et al. 2007; Vrana et al. 2005) and also is associated with logistical and practical difficulties including transportation, filtration, extraction, and storage. Moreover, grab sampling as a way of monitoring pesticide pollution in aquatic environments cannot encompass all of the changes in pollutant concentrations (Ahrens et al. 2015). In other words, determining the dynamic status of pollution accurately during low and high flows is not entirely feasible with grab sampling. Consequently, the outcomes do not directly relate to the average load of pollutants (Jordan et al. 2013). In spite of these facts, grab sampling is useful for finding information quickly. However, extending measurements to cover fluctuations in flow and pollutant concentrations requires increasing the sampling frequency and sample numbers, which is expensive and time consuming while the results remain uncertain (Rozemeijer et al. 2010).

Due to the challenges related to grab sampling, the passive sampling technique is considered as a promising alternative method for measuring pollutants in aquatic environments. This method allows the accumulation of contaminants in the samplers, making it possible to determine very low concentrations of contaminants. Passive sampling provides means for continuous water quality monitoring from short term to long term (a week to some months) and allows determining time-weighted average (TWA) of contaminant concentrations (Ahrens et al. 2015). The chemical potential discrepancy between passive sampler media and the dissolved pollutants in the aquatic phase causes a partitioning of contaminants between water and the sampler (Allan et al. 2009). Moreover, in comparison with organisms, which undergo biotransformation and changing physiological conditions, the uptake of pollutants using passive samplers is more feasible (Smedes and Booij 2012). These features of passive sampling facilitate chemical examination of surface and other water bodies and provide an alternative approach to biomonitoring (Fox et al. 2010; Meyn et al. 2007; Munoz et al. 2010; Wille et al. 2011).

There are various kinds of non-polar passive samplers, which have been used for evaluating organic contaminants in aquatic environments (Brockmeyer et al. 2015). Passive samplers trap the pollutants in a kinetic or equilibrium diffusion, in which the whole process including selective analyte, isolation, and pre-concentration occurs simultaneously (Vrana et al. 2005). The mass transfer of an analyte—an organic or inorganic compound—proceeds until the equilibrium phase occurs or the sampling is finished (Górecki and Namieśnik 2002). Silicone rubber (SR) sheets and Speedisk samplers were selected in this study for the determination of organochlorine pesticides because silicone rubber samplers for more hydrophobic compounds and Speedisk samplers for more hydrophilic compounds are ideal samplers that have some advantages such as simple construction, robust for installing in the rivers or the Lake, cheap, and commonly available (Smedes et al. 2010).

The Lake Naivasha catchment is a major agricultural areas in Kenya, and because of dense agricultural activity and population, there is a high demand for pesticides. Although there was a decline in organochlorine pesticide imports into the country, there is still risk of these pesticides’ application in agricultural areas. Therefore, a monitoring plan for pesticide residue pollution in aquatic environments is necessary to evaluate their potential risk on ecosystems and humans in general. The aim of this research was to gain understanding in the organochlorine pesticide residue pollution and their spatial variation in the Lake Naivasha catchment using passive sampling techniques.

## Materials and methods

### Study area

**Fig. 1:**
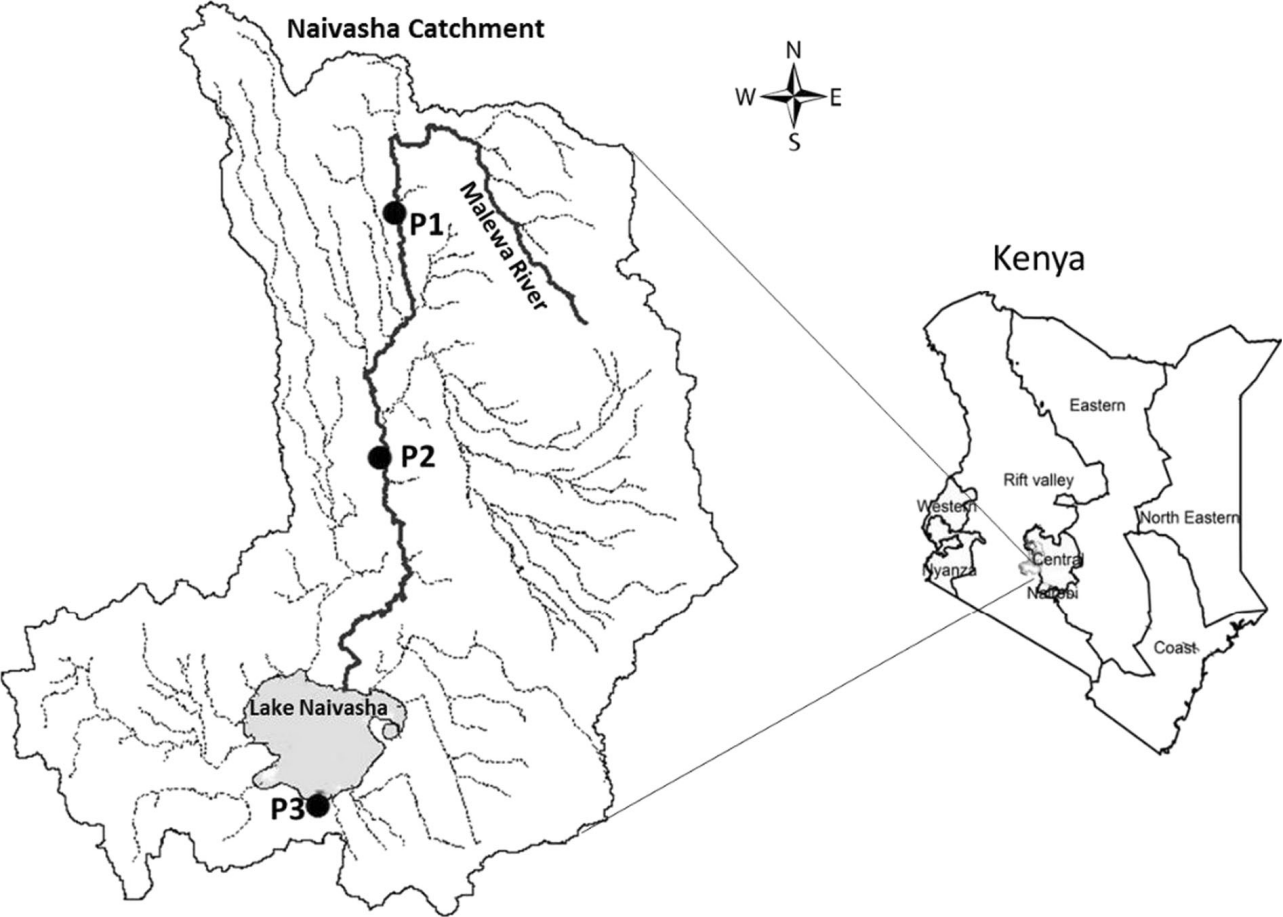
The study area and locations of passive samplers (P1, P2, and P3 are Upper Malewa, Middle Malewa, and the Lake sites, respectively)

Lake Naivasha catchment is located in the eastern part of the Rift Valley region in Kenya with an area of about 3400 km^2^. The eastern rift has a tropical climate with two dry and two rainy seasons. The upper and middle parts of the catchment are mostly subjected to smallholder mixed farming for producing various crops. Moreover, there are various local dwellings in villages and towns all around the catchment that can influence the rivers and the Lake water quality located in the lower catchment. Input from upstream into the Lake includes water from the Malewa, Karati, and Gilgil Rivers plus surface runoff that drains from the catchment and reaches the Lake. But as the Malewa River accounts for approximately 80% of the inflow into Lake Naivasha, the samplers were installed in the Upper Malewa, Middle Malewa River, and the Lake (Fig. 1).

### Sampler preparation and installation

Large AlteSil SR sheets were cut into pieces with 55 × 90 × 0.5-mm dimensions and about 100 cm^2^—both sides—surface area. The SR samplers were pre-cleaned in Soxhlet apparatus with ethyl acetate for at least 100 h to remove all chains of oligomers. Then, they were air dried and spiked with performance reference compounds (PRCs). Based on Smedes and Booij (2012), the SR samplers need to be spiked with at least six PRCs that have a sampler-water partition coefficient (logKpw) between 3.5 and 5.5 as well as a PRC that is rarely depleted (logKpw > 6) and a completely depleted PRC (logKpw < 3.3) for modeling water sampling rate and the concentration of the pollutants. Therefore, the applied PRCs in the SR samplers were BIP-D10, PCB001, PCB002, PCB003, PCB010, PCB014, PCB030, PCB050, PCB021, PCB104, PCB055, PCB078, PCB145, and PCB204. Then, the prepared SR samplers were kept in air-tightened amber glass bottles in the freezer (− 20 °C) until installation.

Speedisk extraction samplers, H_2_O-philic DVB low capacity (0.6 g) produced by Avantor, were also used for more hydrophilic substances. The Speedisks were conditioned by eluting them slowly with 15 mL dichloromethane (HPLC grade, 99.9%), 10 mL acetone (HPLC grade, 99.5%), and 20 mL distilled water, sequentially. They were then stored in a bottle of purified water and stored at + 4 °C until deployment.

**Fig. 2:**
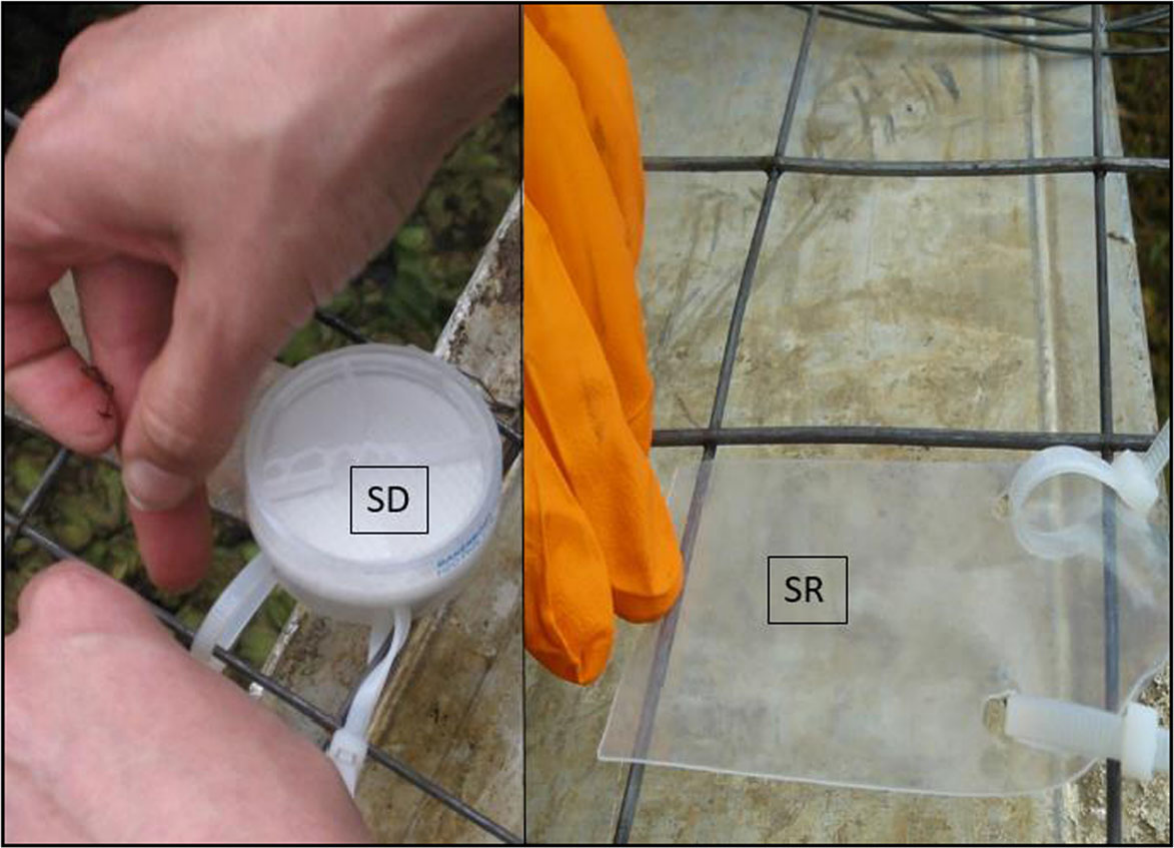
Mounting Speedisk (SD; *left*) and silicone rubber sheet (SR; *right*) passive samplers for deployment

At the sampling sites, including two sites in the Malewa River and one site in the Lake Naivasha as represented in Fig. 1, three sets of silicone rubber sheets and three sets of Speedisk passive samplers were installed for monitoring the concentrations of α-HCH, β-HCH, γ-HCH, δ-HCH, heptachlor, aldrin, heptachlor epoxide, α-endosulfan, pp-DDE, endrin, dieldrin, β-endosulfan, pp-DDD, endrin aldehyde, pp-DDT, endosulfan sulfate, and methoxychlor in the Malewa River and the Lake Naivasha. Both the Speedisk and silicone rubber sheet samplers were mounted on metal wire mesh (Fig. 2) and immediately deployed in water. Additionally, one sampler was exposed to the air while installing the samplers, as reference sampler. Passive samplers were deployed in the water for 1 month from 20 June to 20 July 2016, during the long rainy season when most of the agricultural activity and use of pesticides occur. After 1 month, the samples were collected from the sampling sites. As they were covered by some fouling or algae, they were cleaned using a pre-treated scourer (washed and rinsed with methanol and water) and the water of the same sampling site. Then, the samplers were kept in a cool box (about 5 °C) during transfer and at − 20 °C in the laboratory till treatment and analysis (Monteyne et al. 2013; Smedes and Booij 2012).

### Extraction and analysis

Various solvents such as acetonitrile, hexane, acetone, dichloromethane, and methanol were used to extract the non-polar contaminants from the samplers. The solvents were all of HPLC grade (> 99% purity) in order to extract the studied OCPs. All of the procedures for both the exposed passive samplers and the blanks (control samplers) such as sampler extraction by Soxhlet apparatus, cleanup, concentration, and instrumental analysis were done according to the guidelines by Smedes and Booij (2012) and standard laboratory methods. After solvent extraction from the sampling media, the residues were determined using a gas chromatograph (Agilent 6890N) in combination with an electron capture detector (Agilent μECD) and an auto sampler (Agilent 7683 Series injector) for the OCPs (in Department of Chemistry at University of Nairobi, Kenya), and a gas chromatography (Agilent 7890A) coupled with a mass spectrometer (Agilent 7000 Series Triple Quadrupole MS detector) that had a possibility to measure with an MSMS method for the PRCs in Deltares (TNO laboratory, Netherlands). The temperature program of GC-μECD was set as initially 90 °C (3 min), then 90 to 200 °C (at 30 °C/min and hold time of 15 min), and 200 to 275 °C (at 30 °C/min and hold time of 5 min). The carrier gas was helium and nitrogen was used as make-up gas with a continuous stream of 2 mL/min. The injection mode was pulsed-splitless with a volume of 1 μL. The column was a DB-5 (Agilent, USA) with length of 30 m, internal diameter of 0.32 mm, and film thickness of 0.25 μm. The calibration of the machine was done using the standards of organochlorine pesticides (purity of more than 99%) in 10 concentration levels of 1, 5, 10, 50, 100, 200, 400, 600, 800, and 1000 μg/L. Finally, the quality control of the results was done by triplication for all the samples, and determination of recovery rates from blank treatments. The column of the GC-MS was also DB-5 (length 30 m, ID 0.25 mm, film 0.25 μm). The temperature program was set as initially 70 °C for 1 min, then ramp 1 increase 20 °C/min to 120 min, hold time 0 min; ramp 2 increase 6 °C/min to 250 °C, hold time 0 min; and ramp 3 17.5 °C/min to 300 °C, hold time 2.48 min. The low detection limit was also 1 μg/L for all the PRCs.

### Calculations

The amounts of PRC fraction (*f*_exp_) indicates sampling rate and was estimated as

1 $$ {f}_{exp}=\frac{N_t}{N_0} $$

where *N*_*t*_ and *N*_0_ are the PRC amounts (ng) in the exposed and the reference samplers, respectively. Booij and Smedes (2010) showed that *f* is a continuous function of Kpw and the sampling rate (*R*_*s*_):

2 $$ {f}_{cal}={e}^{\frac{-{R}_st}{K_{pw}m}} $$

where Kpw is the sampler-water partition coefficient (L/kg), *R*_*s*_ is the sampling rate (L/day), *m* is the sampler weight (kg), and *t* is the exposure time (days). Rusina et al. (2010) demonstrated that sampling rate was a function of the hydrodynamic situation and the sampler surface area as well as the PRC molar mass (M). Therefore, their proposed Eq. (3) was used to demonstrate the relationship between these factors:

3 $$ {R}_s=\frac{FA}{M^{0.47}} $$

The sampling rate was estimated by combining Eqs. (2) and (3) and fitting the retained fraction and KpwM^0.47^ using a solver package. The non-linear least squares (NLSs) method, which takes all of the PRCs into account, was applied for this aim (Booij and Smedes 2010).

Booij et al. (2007) showed that the amount of a target compound in the sampler can be presented as

4 $$ {N}_t={C}_w.{K}_{pw}m\left[1-\mathit{\exp}\left(\frac{-{R}_st}{K_{pw}m}\right)\right] $$

Therefore, by adjusting Eqs. (3) and (4), the concentration of compounds was determined as

5 $$ {C}_w=\frac{N_t}{K_{pw}m\left[1-\mathit{\exp}\left(-\frac{FAt}{M^{0.47}{K}_{pw}m}\right)\right]} $$

Finally, the standard deviations (SDs) of the sampling rates as well as the pesticide concentration were calculated and included to the results. The statistical analysis of one-way ANOVA was also applied to explore the differences of total OCPs between the sampling sites at 95% confidence. This examination determined the spatial variation from the upper catchment to the Lake for the results of both kinds of passive samplers.

## Results and discussion

Frequent measurements of different parameters at the sampling sites during sampler exposure time are presented in Table 1. It was found that the average acidity of water in the Malewa River and the Lake was between 7 and 7.8, and no remarkable difference was found. Moreover, the effect of water temperature on sampling rate has been studied by Booij et al. (2003), and they showed that sampling rate at 30 °C was three times more than 20 °C. This issue demonstrates the relations between water temperature and up taking the contaminants. However, by calculating the sampling rates, the effect of different factors (e.g., oxygen saturation, salinity, conductivity, temperature, pH) on the sampler performance is taken to account.

**Table 1:** Physicochemical properties of water at the sampling sites

Location/parameter		pH	*T* (^∘^C)	EC (μS/cm)	Sal. (‰)	Sat. O_2_ (%)
The Lake	Average	7.8	20.1	352.2	0.11	87.1
	Minimum	6.8	18.6	309.0	0.10	53.7
	Maximum	8.6	21.0	366.0	0.12	104.8
Middle Malewa	Average	7.4	16.3	147.6	0.05	103.6
	Minimum	6.7	15.0	111.0	0.04	100.3
	Maximum	8.6	18.0	170.0	0.06	106.4
Upper Malewa	Average	7.7	16.3	150.4	0.05	103.2
	Minimum	7.0	15.6	120.0	0.04	101.7
	Maximum	8.9	17.2	174.2	0.06	105.0

*EC* electrical conductivity, *Sal.* salinity, *Sat.O*_*2*_ oxygen saturation

The results of analysis showed that after 30 days of passive sampler deployment, the average of minimum remaining PRCs on the silicone samplers was 4.7% (± 4.1 SD) for BCP-d10 and the maximum average was 97% (± 7.4 SD) for PCB204. The amounts of remaining PRCs with a logKpw of less than 4.2, such as PCB001 and BIP-D10, occurred on less than 20% of the samplers. The PRCs of PCB014 and PCB104 with a logKpw of more than 5.1 showed a variation of 73% (± 16.7 SD) to 102% (± 1.1 SD), which was in agreement with the results of the study by Monteyne et al. (2013). They indicated that the dissipation of more than 80% and less than 20% of the PRCs leads to difficulties in determining the initial and the final ratio of the PRCs on the samplers. Therefore, it could be concluded that the PRCs with a logKpw of 4.2 (PCB002) to 5.2 (PCB030) would be the most appropriate ones for calculating sampling rate. Moreover, as was concluded in other literature (Allan et al. 2009; Monteyne et al. 2013), the transition between linear and equilibrium phases occurred for the PRCs with logKpw between 4.2 and 5.2. Therefore, PCB010 and other compounds with this range of logKpw were still releasing and the sampling was continued.

**Fig. 3:**
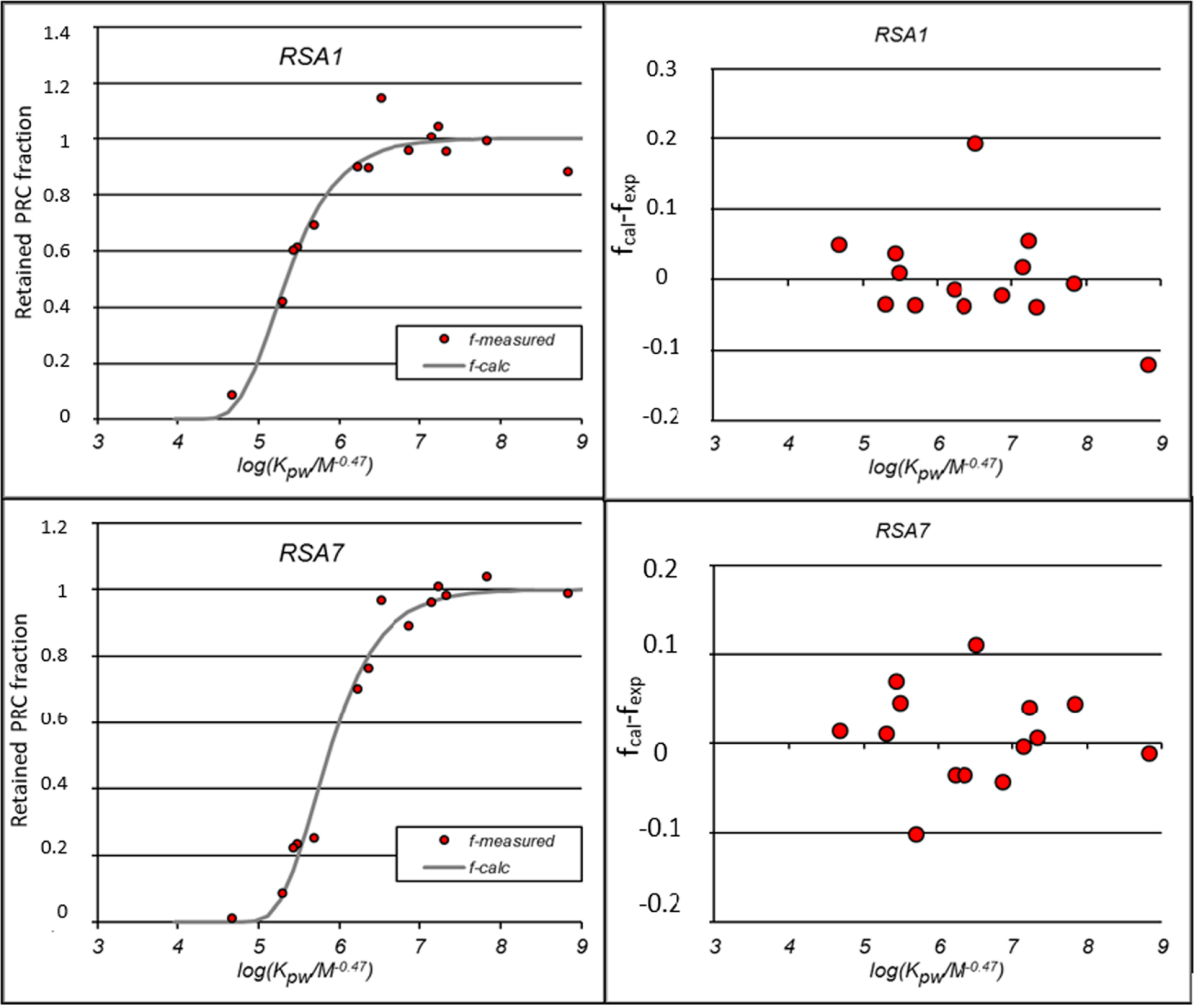
Example diagram of logKpw versus retained PRC fractions (*left*) and difference of calculated and measured (calc.) (*right*). The drawn line represents the best non-linear square for two example sites. RSA1 and RSA7 are example samples in the Lake and in Malewa River, respectively

The results of NLS model showed that there was a good fit between the measured and calculated PRC fractions (Fig. 3). Inclusion of a PRC with a low logKpw such as BIP-D10, which has a logKpw of 3.6, and PCB204, which has a high logKpw of 7.6, as well as other PRCs within this range led to a sigmoid trend among the retained fractions and log(Kpw.M^0.47^). With this approach, the results showed a minimum sampling rate occurring in the samplers that deployed in Lake Naivasha with 1.9 (± 0.4 SD) L/day and a maximum rate at the Middle Malewa river site with 13.1 (± 1.7 SD) L/day. The average sampling rate at the Upper Malewa river site was 6.2 (± 0.7 SD) L/day, an intermediate result compared to other sites. Silicone sheets have been used mostly for monitoring the PAHs and PCBs in marine aquatic environments. However, comparing the results of this study with other literature showed that these results were comparable with the study by Harman et al. (2009), who reported sampling rates between 4.1 and 14.8 L/day for a duration of 6 weeks.

The data of sampling rates were used to determine the pesticide concentrations in the water (Cw). This approach allowed the use of passive sampling technique to monitor non-polar compounds. Calculating the concentration of OCPs with SR samplers showed that the total amount of α-HCH, β-HCH, γ-HCH, δ-HCH, heptachlor, aldrin, isodrin, heptachlor epoxide, α-endosulfan, pp-DDE, endrin, dieldrin, β-endosulfan, pp-DDD, endrin aldehyde, pp-DDT, endosulfan sulfate, and methoxychlor at the Lake site was the highest with a total amount of organochlorine pesticide residue (∑OCPs) of 81 (± 18.9 SD) μg/L. This total amount was 71.5 (± 11.3 SD) and 59 (± 12.6 SD) μg/L for the Middle Malewa and the Upper Malewa river sites, respectively. The reason of reporting the results as a summation of OCPs is that the individual concentrations of the OCPs were mostly low ranging from below detection limit to 56 μg/L.

**Fig. 4:**
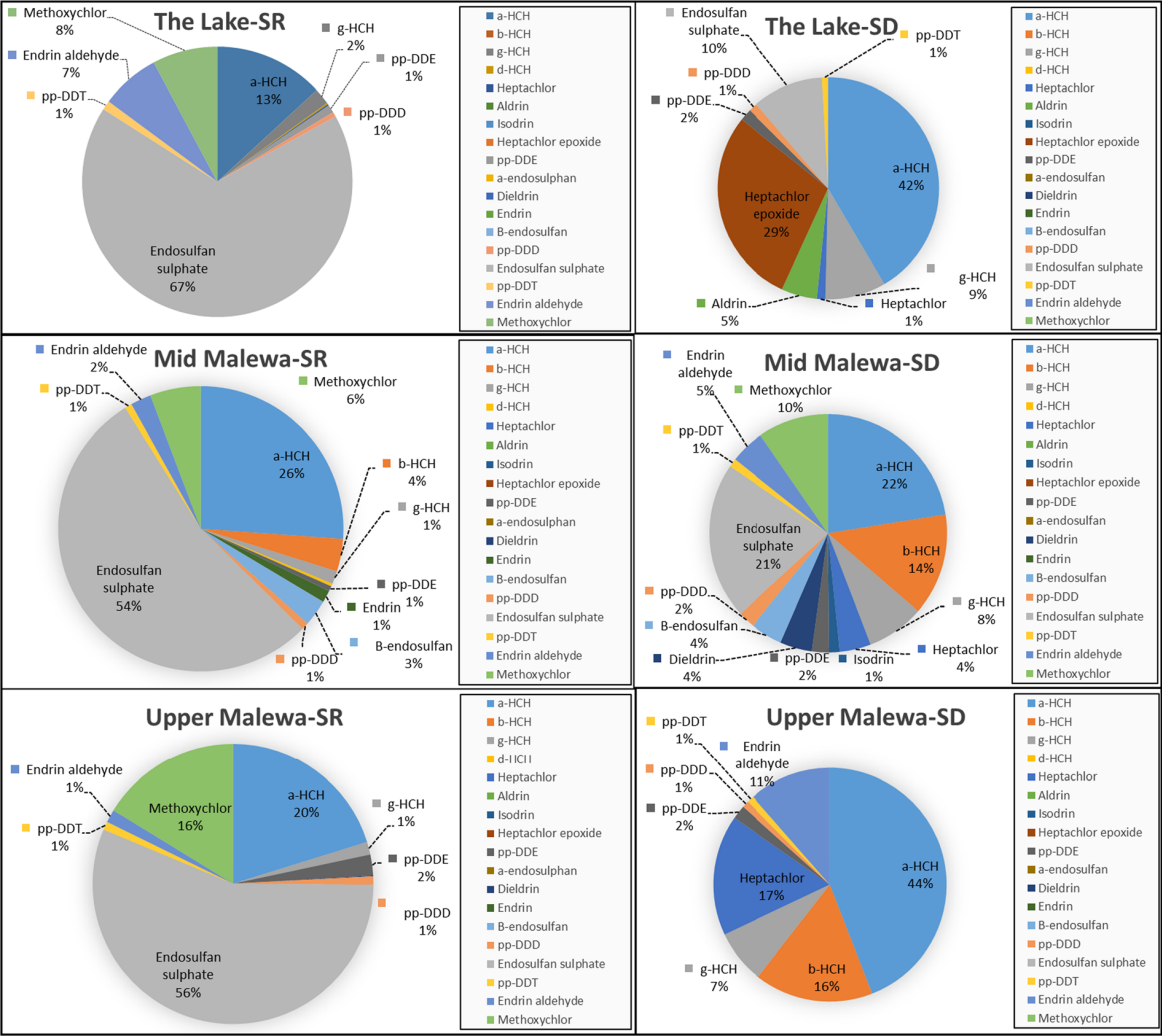
Distribution of organochlorine pesticide residues on the silicone rubber (SR) and Speedisk (SD) passive sampling media at the three sampling sites (based on June–July 2016 sampling campaign data)

The variation in pesticides found at the three sampling sites using SR samplers was also investigated to evaluate possible differences in pesticide residue occurrence in different parts of the catchment (Fig. 4). Although there was an increasing trend from the Upper Malewa to the Lake, the results of one-way ANOVA for the means of the three sampling sites using SR samplers showed that there was not a significant spatial variations (*P* > 0.05). Apart from application of pesticides in the down part of the catchment, as the hydrological stream flow and suspended sediment transport processes is the main reason for agrochemical movement from the upper part of the catchment to the down part, the increasing concentration gradient to the Lake could be due to the effect of downstream transport and accumulation of pesticides. The results showed that α-HCH, endosulfan sulfate, and methoxychlor formed the most prevalent pesticide’s residues. They were detected in almost all of the samplers, and their concentrations generally increased from the Upper Malewa to the Lake site. Based on the results of SR samplers, endosulfan sulfate formed the largest component of pesticide residue in the study area with concentrations of 56 (± 18 SD), 39.3 (± 29.3 SD), and 34.2 (± 11.8 SD) μg/L in the Lake Naivasha, Middle Malewa river, and Upper Malewa river sites, respectively, that accounted, respectively, for 69, 55, and 58% of ∑OCPs in these sites. The second major pesticide residue found on the SR samplers at all of the sites was α-HCH. The concentration of this pollutant varied from 19.3 (± 6.7 SD) μg/L at the Middle Malewa river site (27% of the ∑OCPs) to the amount of 11.3 (± 4.8 SD) μg/L at the Lake site. α-HCH is an isomer of hexachlorocyclohexane (HCH) that has different isomers, and the main ones are α-HCH, β-HCH, γ-HCH, and δ-HCH. All of these isomers are insecticides that are mostly used on fruit, vegetables, and animals. α-HCH is by-product of lindane, but due to the persistence in environment and bioaccumulation, it has been classified as persistent organic pollutant (POP) by Stockholm Convention on Persistent Organic Pollutants in 2009 (ATSDR 2005). Methoxychlor was also found in the SR samplers at all sampling sites. It is an insecticide that has a wide range of application for controlling the insects on crops, livestock, and homes. It dissolves in the water or evaporates into air very rarely, and once it reaches the ground, it sticks to the soil particles that can be transported to water bodies by runoff. The process of degradation in the environment is slow and may take several months (ATSDR 2002a). The ratio of other pesticides occurred in very low percentages. DDT, for instance, accounts for a very low percentage (1–2%) of residue, and this finding is in agreement with previous studies, indicating that the use of this pesticide has significantly decreased (Gitahi et al. 2002). Although in very low concentration, endrin aldehyde was another pesticide that was found in the SR samplers at all of the sites. Comparing the results from the SR samplers at different sites showed that the Middle Malewa river site had most different kinds of applied pesticides. In addition to the mentioned pesticides that were found at the Lake and Upper Malewa river sites, β-HCH, endrin, β-endosulfan, pp-DDD, and pp-DDE were found at the Middle Malewa river site. pp-DDD and pp-DDE, occurring with a concentration of less than 1 μg/L (almost 1% ratio of ∑OCPs) at the Middle Malewa river site, are the metabolite of DDT, which may originate from pesticide application in the past still present in the environment. It is noticeable that DDT/(DDD + DDE) ratio is an indication of DDT application history that the amount of less than 1 means that there might not be current input of the parent DDT into the study area and vice versa (Gbeddy et al. 2012). The results showed that this ratio was less than 1 for the sampling sites.

Considering the sampling rates of Speedisk samplers and the total of pesticides taken up by these samplers, the maximum amount of pesticides was ∑OCPs = 34.2 (± 6.5 SD) μg/L that was found at the Lake Naivasha site. The amounts of ∑OCPs from the Speedisk samplers at Middle Malewa and Upper Malewa sites were 31.3 (± 1.8 SD) and 28.2 (± 4.2 SD) μg/L, respectively. Based on the one-way ANOVA results of Speedisks for exploring the spatial variations of the OCPs at the sampling sites, these amounts were not significantly different (*P* > 0.05).

Evaluating pesticide variation in the studied area by Speedisk samplers also demonstrated that α-HCH occurred at all of the sampling sites (Fig. 4). The Lake site, with a concentration of 16.1 (± 5.1 SD) μg/L, which is equivalent to 47% of ∑OCPs, revealed the highest measured amount. The concentration of this pesticide’s residue was 13 (± 1.9 SD) μg/L in the Upper Malewa river and decreased to the 5.9 (± 1.8 SD) μg/L in the Middle Malewa river(19% of ∑OCPs). Although the Middle Malewa and Upper Malewa river sites were situated in the same river, the sites were placed far apart to discover the effect of the surrounding agricultural areas of the sampling sites on the pollution situation of the Malewa River. The selected site location in the Lake Naivasha was also at the opposite side of the Lake to the Malewa river estuary, in order to minimize the effect of Malewa River on the Lake Naivasha sampling site. Therefore, the results of each of the sites can be said to be mostly related to the pollution in adjacent areas. Moreover, although the interview with the farmers about the pesticides use for controlling any kind of diseases in their products (e.g., cabbage, tomato, potato, maize) did not show any OCP application, the results of sampler analysis demonstrated the OCP residues in the sampling sites. Nearly all of the explored HCH isomers were found in the Middle and Upper Malewa Rivers. It is noticeable that some of the OCPs such as α-HCH, β-HCH, γ-HCH, δ-HCH, pp-DDT, pp-DDE, and pp-DDD that are metabolites of HCH and DDT can remain in the environment for an extremely long time by accumulating in different environmental compartments. For instance, when DDT is broken down by microorganisms or under environmental condition, DDE and DDD are produced which are similar to their parents. The residue of these chemicals that are not dissolved easily in water can stick to soil particles and remain in environment up to 15 years (ATSDR 2002b).

Passive samplers accumulate pesticide residues from the water during deployment; therefore, they may be more useful than grab sampling for finding OCPs. Moreover, as the behavior of the passive samplers for accumulating the contaminants mimics the bioaccumulation by organisms (e.g., uptake of pesticides by aquatic biota like fish), the outcomes of passive sampling studies are more comparable with biomonitoring ones (Smedes and Booij 2012). Therefore, the results of this study can be compared with the study by Gitahi et al. (2002) that explored pesticide contamination in water resources of Naivasha using fish samples. Their study about organochlorine pesticide pollution in various species of fish, water, and sediment samples in Lake Naivasha demonstrated different levels of lindane, dieldrin, β-endosulfan, and aldrin in the fat of fish. Their results showed a technical use of these pesticides in the studied area, which would agree with the findings of this study.

Based on the report of the Pest Control Products Board of Kenya PCPB (2008), import and use of many of the studied pesticides have been discontinued. However, different studies (Gitahi et al. 2002; Onyango et al. 2014) indicated application of these pesticides in the Lake basin. Lindane was a commonly used pesticide in Kenya. It was used as insecticide and for seed dressing. HCH and its isomers are the main pesticide residues found in the Speedisk samplers. These results seem to indicate the use of HCH and its isomers in the Lake Naivasha catchment. Endosulfan sulfate is an oxidation product of endosulfan, which has a high acute toxicity and can be potentially bioaccumulated. Because of the threats of endosulfan and its isomers (α-endosulfan, β-endosulfan, and endosulfan sulfate) to human health and the environment, there was a global ban on its application under the Stockholm Convention. Based on Camacho-Morales and Sánchez (2015), the estimated half-life time of these chemicals (endosulfan and endosulfan sulfate) can vary from 9 months to 6 years. However, endosulfan sulfate was found in the SR samplers at all of the sampling sites.

**Fig. 5:**
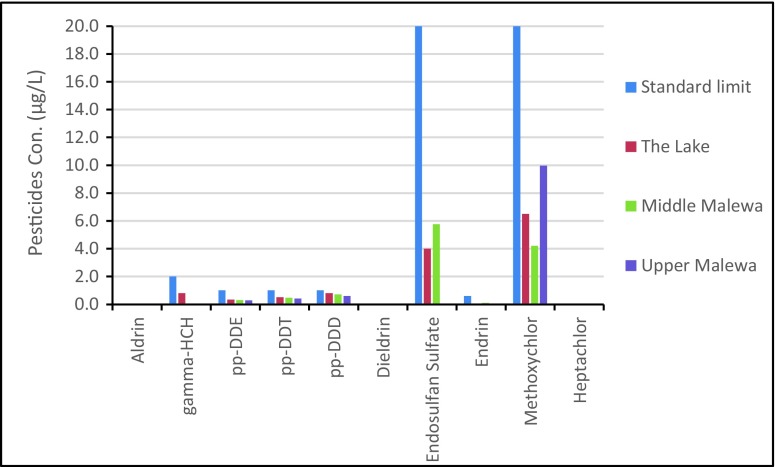
Comparison of measured pesticide residue concentrations at the three sites studied to WHO drinking water standards

Comparing the pollution levels of pesticides in the water for different sampling sites with the drinking water standards criteria (WHO 2011) showed that the concentrations of all the studied pesticides were below the WHO drinking water standard and limits (Fig. 5). Results of this study are in agreement with another recent pesticide residue study in the Lake basin by Onyango et al. (2015). They reported on 4,4-DDT, 2,4-DDE, 4,4-DDD, γ-HCH, α-HCH, and aldrin contamination in the aquatic environment of the Lake Naivasha catchment and concluded that there was no potential effect of these pesticides on human health in drinking water. However, because of bioaccumulation of pesticides in aquatic organisms and the risk of entering the higher food web and chain, the concentration of pesticide residues, even in low concentrations, needs to be monitored continuously.

## Conclusions

This study investigated organochlorine pesticide residues in surface water resources on Naivasha, Kenya using passive sampling techniques. Analysis of OCP concentrations showed that the total amounts at the Lake site was highest. This could be due to the fact that the Lake was the final accumulation site for runoff and suspended sediments from the basin. The ∑OCPs for the Middle Malewa and the Upper Malewa river sites represented the second and third levels, respectively. The results showed that endosulfan sulfate was the main pesticide residue found at all of the sampling locations. The results from the SR samplers showed that there was also contamination by α-HCH, endrin aldehyde, and methoxychlor at all of the sites. Finally, it was concluded that continuous monitoring of OCPs and other pesticides using passive sampling was a very useful technique that could contribute to environmental monitoring and pollution assessment of water resources in the studied area.
